# Quantification of Resection Margin following Sublobar Resection in Lung Cancer Patients through Pre- and Post-Operative CT Image Comparison: Utilizing a CT-Based 3D Reconstruction Algorithm

**DOI:** 10.3390/cancers16122181

**Published:** 2024-06-10

**Authors:** Yu-Hsuan Lin, Li-Wei Chen, Hao-Jen Wang, Min-Shu Hsieh, Chao-Wen Lu, Jen-Hao Chuang, Yeun-Chung Chang, Jin-Shing Chen, Chung-Ming Chen, Mong-Wei Lin

**Affiliations:** 1 Department of Biomedical Engineering, College of Medicine and College of Engineering, National Taiwan University, Taipei 106, Taiwan; r09528013@ntu.edu.tw (Y.-H.L.); f04548034@ntu.edu.tw (L.-W.C.); d04548013@ntu.edu.tw (H.-J.W.); 2 Department of Pathology, National Taiwan University Hospital and National Taiwan University College of Medicine, Taipei 100, Taiwan; mshsieh065@gmail.com; 3 Department of Surgery, National Taiwan University Hospital and National Taiwan University College of Medicine, Taipei 100, Taiwan; i2363160@gmail.com (C.-W.L.); renhao912@hotmail.com (J.-H.C.); chenjs@ntu.edu.tw (J.-S.C.); 4 Department of Medical Imaging, National Taiwan University Hospital and National Taiwan University College of Medicine, Taipei 100, Taiwan; ycc5566@ntu.edu.tw

**Keywords:** computed tomography, lung cancer surgery, resection margin, sublobar resection, image registration, 3D reconstruction, subvascular tree matching, discontinuous deformation field

## Abstract

**Simple Summary:**

This study introduces a CT-based 3D reconstruction algorithm for measuring resection margins post sublobar resection in lung cancer patients. It aims to enhance accuracy and reproducibility, addressing the limitations of gross margin measurement. The findings show feasibility with a target registration error of <2.5 mm. This approach could revolutionize lung cancer treatment, facilitating prompt identification of patients with inadequate resection margins for timely post-operative adjuvant therapies.

**Abstract:**

Sublobar resection has emerged as a standard treatment option for early-stage peripheral non-small cell lung cancer. Achieving an adequate resection margin is crucial to prevent local tumor recurrence. However, gross measurement of the resection margin may lack accuracy due to the elasticity of lung tissue and interobserver variability. Therefore, this study aimed to develop an objective measurement method, the CT-based 3D reconstruction algorithm, to quantify the resection margin following sublobar resection in lung cancer patients through pre- and post-operative CT image comparison. An automated subvascular matching technique was first developed to ensure accuracy and reproducibility in the matching process. Following the extraction of matched feature points, another key technique involves calculating the displacement field within the image. This is particularly important for mapping discontinuous deformation fields around the surgical resection area. A transformation based on thin-plate spline is used for medical image registration. Upon completing the final step of image registration, the distance at the resection margin was measured. After developing the CT-based 3D reconstruction algorithm, we included 12 cases for resection margin distance measurement, comprising 4 right middle lobectomies, 6 segmentectomies, and 2 wedge resections. The outcomes obtained with our method revealed that the target registration error for all cases was less than 2.5 mm. Our method demonstrated the feasibility of measuring the resection margin following sublobar resection in lung cancer patients through pre- and post-operative CT image comparison. Further validation with a multicenter, large cohort, and analysis of clinical outcome correlation is necessary in future studies.

## 1. Introduction

Lung cancer is one of the leading causes of cancer deaths worldwide. For stage I lung cancer, surgical resection is the treatment of choice. For stage II-IIIA lung cancer, surgical resection is often combined with perioperative medical treatment. For small peripheral lung cancers less than 2 cm, sublobar resection offers considerable advantages, particularly for patients with compromised cardiopulmonary function, making it a more suitable option [1,2,3,4]. However, compared to lobectomy, sublobar resection may be associated with higher rates of tumor recurrence in centrally located lung cancer, with inadequate resection margin distance being a major contributing factor [5,6,7]. According to the National Comprehensive Cancer Network guidelines for non-small cell lung cancer, a resection margin distance of greater than or equal to 2 cm or tumor size is recommended for sublobar resection [8]. Ensuring an adequate resection margin distance may significantly reduce local recurrence rates [5,6,7]. However, conflicting opinions exist regarding the impact of resection margin distance on local recurrence rates [9,10,11]. The lack of consensus may be attributed to the reliance on subjective measurements by surgeons or pathologists, using direct visual observation of surgical specimens. Such measurement methods may be unreliable and prone to errors, including inconsistent lung collapse, unclear tumor margins, variations in the angle of incision, and measurement of distances that are not the closest from the tumor margin to the resection margin.

Therefore, this study aims to revisit this issue using medical imaging analysis methods and to develop relevant processes and techniques to provide a new measurement approach for greater accuracy and stability. The object of this study is to develop an objective measurement method, the CT-based 3D reconstruction algorithm, to quantify the resection margin following sublobar resection in lung cancer patients through pre- and post-operative CT image comparison.

## 2. Materials and Methods

### 2.1. Data Information

**Figure 1:**
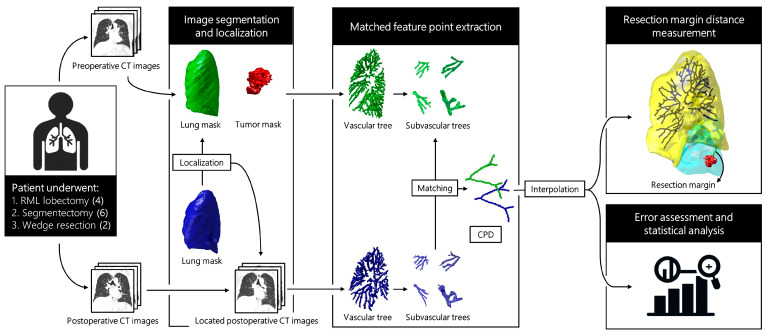
Framework of this study. CPD, coherent point drift; CT, computed tomography; RML, right middle lobe.

The data utilized in this study were collected from National Taiwan University Hospital between January 2017 and December 2019. The dataset consisted of 12 cases involving patients clinically diagnosed with lung cancer who underwent either lobectomy or sublobar resection, encompassing 4 right middle lobectomies, 6 segmentectomies, and 2 wedge resections. Right middle lobectomy was specifically chosen due to its minimal deformation post-resection, serving as mutual verification between the expected surgical resection range and algorithm reconstruction results. The imaging modality selected for the study was thoracic computed tomography (CT) scans. For lung cancer patients, a set of images for lung cancer screening are taken pre-operatively, and another set of post-operative CT scans are performed within three to six months after surgery for follow-up. Thus, each lung cancer patient has two sets of thoracic CT scan images, one pre-operative and one post-operative. This study aims to reconstruct the surgical resection area, requiring high image resolution. Therefore, we chose thin-slice images with slice thickness ranging from 0.62 to 1.25 mm. This retrospective study was approved by the research ethics committee of NTUH (project approval number: 201712087RIND, approval date: 23 January 2018), and the requirement for informed patient consent was waived. The overall flowchart of this study is illustrated in Figure 1.

### 2.2. Surgical Protocol for Sublobar Resection

At our institute, a lobectomy was considered if the tumor diameter was more than 2 cm or if the tumor was centrally located. Sublobar resection was considered for patients who were older, had compromised lung function, or were at high surgical risk. We measure the resection margin based on gross measurement during the surgery.

Before segmentectomies, surgical planning with pre-operative three-dimensional reconstruction using Synapse 3D (Fujifilm Techno Products Co., Ltd., Tohoku Factory Hanamaki Site, Tokyo, Japan) was carried out to guide the division of the bronchus and vessel to ensure complete tumor resection with adequate margins. For intersegmental plane definition, we use the inflation–deflation method or near-infrared fluorescence imaging with intravenous indocyanine green after division of the target bronchus or pulmonary artery. Lymph node dissection, including N1 and N2 nodes, was performed during and after the anatomical resection.

### 2.3. CT Image Acquisition and Pre-Processing

In this study, chest CT scans were obtained using multi-detector CT scanners (16-, 32-, or 64-detector row) from various manufacturers including Philips (iCT 256 and Ingenuity CT, Amsterdam, The Netherlands), Canon Medical Systems (Aquilion ONE, Otawara, Tochigi, Japan), GE Medical Systems (Revolution CT, Waukesha, Wisconsin, USA), and SIEMENS (Sensation 64 and SOMATOM Definition AS+, Erlangen, Germany). The imaging parameters included a range of detector collimations (0.62–1.25 mm), fields of view (30.4–40.9 cm), beam pitches (0.813–1.200), and beam widths (10–40 mm), with gantry speeds of 0.5 or 0.8 s per rotation. The scans were performed at 120 kVp and 60–649 mA, with reconstruction intervals of 0.39–6 mm and a matrix size of 512 × 512 m.

Both pre- and post-operative thoracic CT scans were included for each case in this research. Given the variation in scan parameters, a resampling method using linear interpolation was implemented. This was crucial to standardize the image sizes to a consistent spatial resolution of 1:1:1 mm, ensuring uniform voxel-to-tissue volume mapping.

### 2.4. Image Segmentation and Localization

Following the pre-processing of pre-operative and post-operative images, segmentation of lungs and tumors was conducted on both pre- and post-operative CT images. Subsequently, the segmented lungs were utilized for initial image registration, aimed at achieving image localization. This part of the workflow is depicted in the left half of Figure 1. Upon obtaining the post-operative images localized based on the pre-operative images, segmentation of the pulmonary vascular and subvascular trees was then performed individually on both pre-operative and localized post-operative images. This part of the process is illustrated in the middle section of Figure 1.

#### 2.4.1. Lung and Lung Tumor Segmentation

To focus on the anatomical structures within the lung regions and assist in preliminary localization, lung masks were initially extracted. This extraction process [12] utilized a 3D Gaussian filter to reduce noise, followed by thresholding at −775 Hounsfield Units and the identification of 3D connected components to delineate the lung tissue.

Delineation of tumor margins was crucial for measuring the resection margin distance. A semi-automatic method [13] was employed. This approach, integrating threshold-based lung wall segmentation [14] with a hybrid level-set method [15], initiated the process. The segmentation was further refined using morphological operations [16] and a Frangi-based approach [17], which were instrumental in removing both small and large vessels, thereby enhancing segmentation accuracy.

#### 2.4.2. Image Localization

During thoracic CT scanning, variations in pre- and post-operative lung image positioning can occur due to differences in scanning equipment and settings. To aid subvascular tree segmentation and matching while standardizing procedures, initial lung mask registration was conducted for localization of lung regions before and after surgery. The ‘imregister’ function in the Image Processing Toolbox of MATLAB version 2020a (MathWorks, Natick, MA, USA) was utilized. Pre-operative lung masks served as fixed images, while post-operative scans were set as moving targets, with affine transformations applied for registration. This method, adhering to default monomodal parameters, corrected scan positioning and respiratory phase discrepancies, ensuring accurate and stable subsequent analysis.

#### 2.4.3. Pulmonary Vascular Tree and Subvascular Tree Segmentation

The proposed method leverages pulmonary vascular tree information as key features for image registration, introducing and defining subvascular trees as sub-units of the vascular tree for matching purposes to obtain distinctive registration features. This approach begins with the segmentation of the vascular tree. The algorithm for pulmonary vessel segmentation in this study utilizes a vessel-enhancing filter based on the Hessian matrix [17] specifically to enhance lung tubular structures. Segmentation is achieved using a threshold at the 95th percentile. To control vessel caliber, images are preprocessed with a Gaussian kernel (σ). Typically, σ is set to 1, 2, 3 mm for detailed vasculature, but inspired by Cazoulat et al. [18], σ was set to 3 mm to capture more representative vascular structures, thus aiding in matching stability.

**Figure 2:**
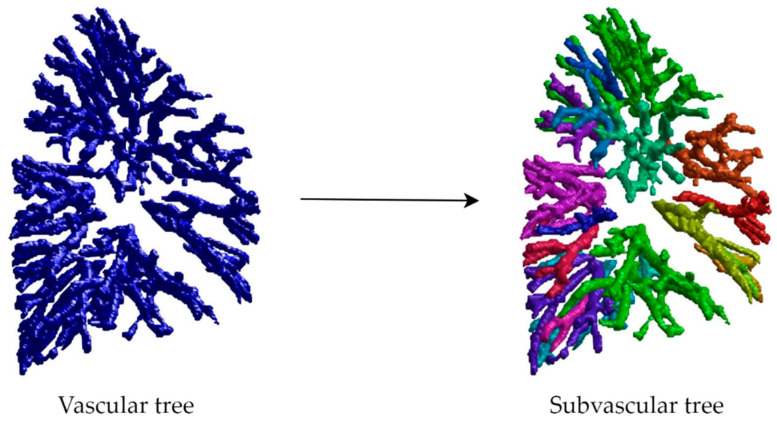
Process of subvascular tree segmentation: The (**left**) image displays the segmented pulmonary vascular tree; the (**right**) image shows the further segmented subvascular tree, with each subvascular tree represented by different colors.

Pulmonary vascular trees were extracted from pre- and post-operative images and masked using the lung regions eroded by 4 mm, excluding main pulmonary arteries and veins. Subvascular trees were then identified using the 26 connected-component technique, with targets over 250 voxels considered individual subvascular trees. The segmentation and definition of these subvascular trees aim to ensure the stability of subsequent matching by utilizing sub-units and regional concepts. The process of subvascular tree segmentation is shown in Figure 2.

### 2.5. Matched Feature Point Extraction

Upon obtaining the segmentation results of subvascular trees before and after surgery, the goal is to match identical subvascular trees and consider points on their centerlines as feature points. Consequently, pairs of corresponding feature points are identified within pre-operative and post-operative images for facilitation in image registration interpolation. This part of the process is illustrated in the middle section of Figure 1.

#### 2.5.1. Subvascular Tree Matching

Subvascular tree matching, a fully automated method in this study, is designed for the matching of subvascular tree structures in post-operative lung imaging. The technique involves adjusting to the changes in the number of subvascular trees due to surgery. Furthermore, the concept of subvascular trees is crucial in addressing the matching challenges posed by complex deformations, making them more manageable.

The matching process is strategically organized into two consecutive phases, with Phase I dedicated to establishing high-similarity matches first. This structured approach leverages the information gathered from Phase I to address the remaining matches in Phase II, ensuring an accurate, automated, and replicable subvascular tree matching process. The detailed steps of Phase I and II are as follows:

- Phase I: Establishing high-similarity matches
  1. This phase begins with the input of two sets of subvascular trees, categorized as pre-operative and post-operative, along with their respective overall vascular trees.
  2. The process involves skeletonizing these trees to define their structures for precise localization.
  3. Using rigid coherent point drift (CPD) [19], the structures (centerline points) are aligned, resulting in a transformation matrix. The primary goal here is to position the pair of subvascular trees. This matrix is then applied to the post-operative trees for alignment with the pre-operative ones.
  4. Surrounding areas (60 × 60 × 60) from both pre- and post-operative trees are cropped to create volumes of interest, which are crucial for the subsequent analysis.
  5. The similarity between the transformed post-operative subvascular trees and their pre-operative counterparts, as well as between the respective surrounding areas, is calculated using the Dice similarity coefficient (Formula (1)). Decision making is based on set threshold values for these similarity coefficients, typically ‘≥0.5’ for target similarity and ‘≥0.2’ for surrounding area similarity.
  6. Based on these thresholds, a decision is made to either confirm the matching of the pre-operative trees or to output a null result in cases of mismatch.

- Phase II: Appending relative positioning for remaining matches
  1. The input for this phase includes an unmatched post-operative subvascular tree, a pre-operative vascular tree, and several matched subvascular tree pairs.
  2. The centroids of these matched pairs are determined to provide reference points for locating potential matches.
  3. The searching center is established by selecting three points closest to the unmatched post-operative tree from the matched post-operative centroids and identifying their corresponding points in the pre-operative set.
  4. A 3 mm morphological dilation is performed on the unmatched post-operative subvascular tree to create a mask for searching potential matches in the pre-operative tree.
  5. The mask is used for image localization around the searching center to identify potential targets in the pre-operative tree.
  6. The similarity between the post-operative tree and these potential pre-operative targets is calculated, incorporating a penalty term (Formula (2)) to avoid incorrect matching, particularly in smaller trees. The target with the highest similarity is selected for matching.
  7. A decision is made based on the similarity measure; if the similarity exceeds a threshold (≥0.3), the corresponding pre-operative target is confirmed as a match. Otherwise, a null result is produced.

The Dice similarity coefficient (*DSC*) is given by the formula:

(1) $$DSC=\frac{\left|V_{pre}\cap V_{post}\right|}{\left|V_{pre}\right|+\left|V_{post}\right|}$$

The penalization term $r^{2}$ is incorporated into the basic Dice coefficient to design the following similarity coefficient:

(2) $$DSCP=\frac{DSC}{r^{2}},r=\frac{V_{1}}{V_{2}}\;\left(V_{1}\geq V_{2}\right)$$

Here, the matching objects are labeled $V_{1}$ and $V_{2}$ based on their volumes, ensuring $r^{2}\geq 1$. This approach allows for the adjustment of the Dice coefficient to impose a penalty.

#### 2.5.2. Feature Point Matching

Following the subvascular tree matching, skeletonization is performed to extract centerlines as feature points, employing boundary expansion and iterative voxel examination to preserve topological integrity [20]. The process ends when no boundary changes occur, concluding with the removal of padding zeros for the skeletonized output.

**Figure 3:**
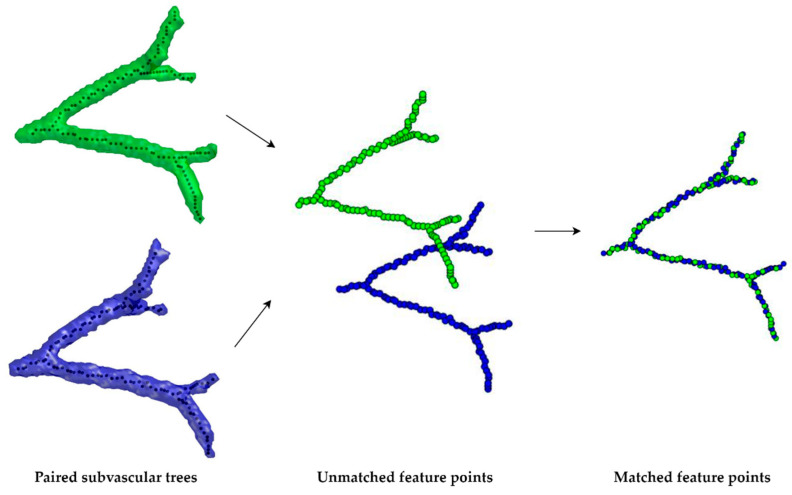
Process of feature point matching: Pre-operative feature points are represented in green, while post-operative feature points are shown in blue. The middle illustration displays the state of feature points before matching, and the right-side image demonstrates the situation after feature point matching has been completed.

For aligning feature points of subvascular trees before and after surgery, the non-rigid coherent point drift (CPD) [19] algorithm is employed, chosen for its robustness and its specific ability to handle the anatomical changes and respiratory phase differences that affect vascular structures. Given that subvascular trees can vary considerably, leading to point cloud outliers, CPD proves effective in managing these outliers, ensuring that the alignment results remain consistent and less affected by variations. This method is particularly effective in achieving a set-to-set point correspondence between each paired subvascular trees, mapping each post-operative point back to its pre-operative counterpart. As such, the post-operative feature points and their mapped counterparts can serve as reference point pairs for subsequent image registration. The process of feature point matching is shown in Figure 3.

### 2.6. Image Registration

After the extraction of the matched feature points, the focus shifts to calculating the displacement field within the image via thin-plate spline transformation [21]. Traditional methods use all available control points to ensure a continuous deformation field, but this can be limiting near surgical sites due to discontinuities from suturing or lung collapse. Inspired by the inverse distance weighting (IDW) interpolation method [22] used in geospatial analysis, our innovative approach selectively uses only a subset of control points. This allows us to intentionally introduce discontinuities around areas such as surgical excisions, mimicking natural irregularities that occur post-operatively.

Control point selection is tailored to address voxels near tumors, anticipating discontinuous deformation. A calculated candidate radius (Formula (3)) identifies potential control points, which are then refined through clustering using the DBSCAN algorithm [23]. This method enables automatic grouping based on displacement trends without pre-setting cluster numbers, focusing on local versus global deformation traits by selecting the most relevant control points. The process incorporates a specific algorithm for control point selection, detailed as follows:

1. The process begins by inputting a voxel point, the tumor’s location, a predefined maximum distance, and a pair of matched feature points.
2. The displacement between each pair of post-operative feature points is determined.
3. A radius for the sphere of interest is calculated, and control points within this radius are selected.
4. The DBSCAN algorithm is applied to these selected control points based on their displacement, resulting in several clusters.
5. The cluster closest to the original voxel point is chosen, effectively balancing local and global deformation characteristics.
6. This selection outputs a pair of control points, marking the end of the control point selection process.

(3) $$R=2\times d_{max}\times exp\left(a\times \left(\frac{d}{d_{max}}-1\right)\right)$$

In the formula, R denotes the candidate radius, d is the distance between a voxel point and the tumor (centroid representative), and $d_{max}$ is the furthest distance from the tumor to the lung’s edge. The adjustable parameter a is set to 3, leading to an exponential growth pattern in the formula y=exp(a(x−1)), keeping y within [0,1]. This setup aims to reflect that voxel points closer to the tumor will show more localized deformation characteristics, whereas points further away will exhibit global deformation traits.

### 2.7. Resection Margin Distance Measurement

Upon completing the final step of image registration, the core objective of the study is addressed: measuring the distance at the resection margin. This involves overlapping the deformed post-operative lung mask, which represents the state potentially resected during surgery, with the pre-operative tumor mask within the same coordinate system. The aim is to reconstruct and predict the surgical resection boundaries. The Euclidean distance between the closest points on the resection and tumor edges is then measured, providing a quantified result for the resection margin distance. This distance is determined by identifying the two closest points between the deformed post-operative lung mask and the pre-operative tumor mask.

### 2.8. Error Assessment and Statistical Analysis

To ensure the accuracy of image registration outcomes, this study employs target registration error (TRE) as the primary evaluation tool. TRE is used to assess the deviation between specific target points’ actual anatomical locations and their corresponding positions on the registered images, typically measured through Euclidean distance. This involves comparing the spatial distance difference between an anatomical point marked on pre-operative images and its deformed position on post-operative images.

For selecting target point pairs, manual marking on pre- and post-operative original images was performed by experienced physicians, focusing particularly on easily identifiable pulmonary vessels or bronchial branch points. This approach ensures that the marked points are clinically relevant and accurately represent the anatomical regions of interest. Considering the potential significant changes in lung structure before and after surgery, the original images were processed with maximum intensity projection (MIP) across five slice levels to more accurately locate and match corresponding target points before and after surgery. Additionally, in selecting target point pairs for TRE, points were chosen distributed throughout the entire lung area, which more comprehensively reflects the accuracy of the image registration across the entire range of the lung regions.

Moreover, the study plans to compare this method with the multi-channel lung registration method based on image features recently proposed by Stavropoulou et al. [24], analyzing both methods through a paired sample t-test. Twenty sets of target points were selected for analysis in both the left and right lungs, including twenty pairs each from non-lesioned lung areas and surgical lung areas.

## 3. Results

### 3.1. Subvascular Tree Matching

#### 3.1.1. Experiment on Lung Regions without Lesions

The study involves utilizing CT images of lung regions without lesions to test the subvascular tree matching algorithm. To mitigate variations in image quality caused by different imaging equipment or scanning parameters, a set of 12 images from the same surgical cases is chosen, focusing specifically on the lung side not affected by surgery (for example, experiments will be conducted on the unaffected left lung when surgery is performed on the right lung).

As shown in Table 1, the average matching completion rate for all 12 cases in the first phase was 67%. Typically, the vascular tree structures of lungs without lesions at two different time points should not vary significantly. However, differences in the respiratory phase of patients during CT scans at different times, the use of different scanners, or variations in scanning parameters must be considered. Even when lung vascular segmentation is performed with the same parameters, the segmentation results can differ, explaining the variance in matching rates between cases. For the first phase, as long as a certain number of matches are achieved, it will not affect the progression of the second phase. The aim of the first phase is to match subvascular tree pairs with higher similarity, providing more information for the second phase to complete the matching of the remaining unmatched subvascular trees.

**Figure 4:**
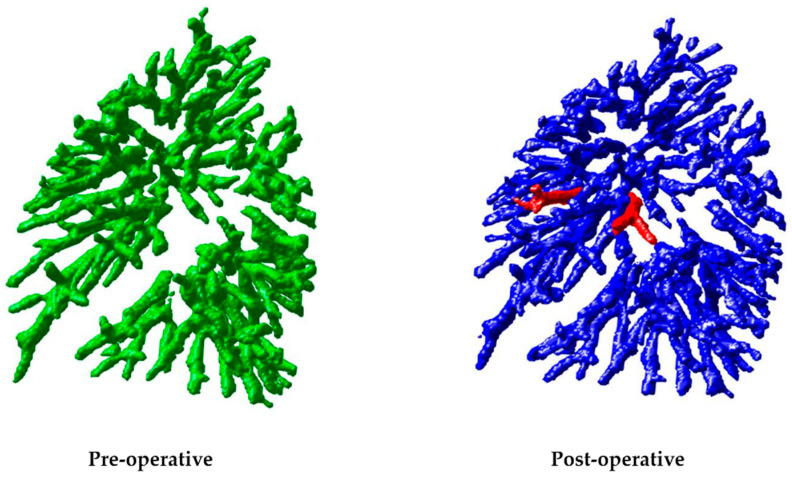
Display of unmatched subvascular trees in case 4: In the image on the (**left**), the pre-operative vascular tree structure is represented in green, while the image on the (**right**) shows the post-operative vascular tree structure in blue. The unmatched subvascular trees are marked in red in the right-hand image.

After completing the first phase, the study moves into the second phase, focusing on matching the remaining subvascular trees. In these 12 cases, except for cases 4, 6, 8, 10, and 12, the rest successfully matched all subvascular trees. However, in cases 4, 6, 8, 10, and 12, one to two sets of subvascular trees remained unmatched. Taking case 4 as an example, as shown in Figure 4, the two unmatched subvascular tree structures were relatively simple. Upon comparing the vascular tree structures before and after surgery, it was noted that similar structures were not present in the pre-operative images, possibly due to differences in image quality affecting the vascular tree structure during segmentation, meaning it did not appear in the pre-operative vascular segmentation and could not be matched. Even though these subvascular trees could not be matched, other successfully matched subvascular trees in the vicinity, with at least 1000 pairs of feature points evenly distributed throughout lesion-free lung regions, provided sufficient corresponding information to complete the final image registration.

#### 3.1.2. Experiment on Surgical Lung Regions

Following the successful experiments in lung regions without lesions, the focus shifts to the core part of our study: the matching experiments of pre- and post-operative subvascular trees in the lungs. Subsequently, subvascular tree matching is conducted on all 12 cases, which include patients who have undergone lobectomy, segmentectomy, and wedge resection, involving various resected lung regions.

As shown in Table 2, the average success rate of subvascular tree matching in the first phase of the 12 cases was 48%, showing a significant gap compared to the 67% in experiments with lung regions without lesions. This result is within expectations, as surgery involves the resection of parts of the lung, leading to greater differences between pre- and post-operative images. Specifically, some vascular tree structures present before surgery were removed and thus not present in post-operative images. This led to the exclusion of subvascular tree pairs that could not be confidently matched in the first phase.

**Figure 5:**
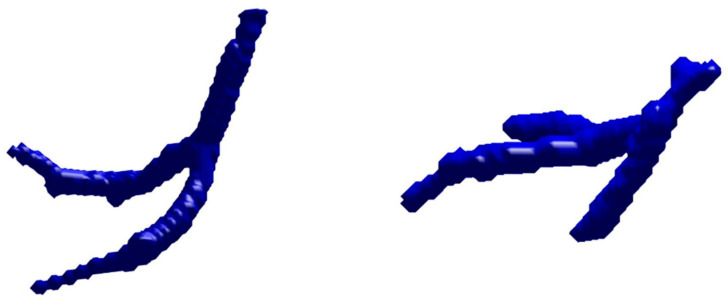
Display of two unmatched subvascular trees in case 6.

In the subsequent content, focus will be on the matching results of the second phase. Compared to experiments with lung regions without lesions, it was observed that in 12 cases, 9 exhibited incomplete matching. Notably, in case 6, the most unmatched sets of subvascular trees, totaling three, were identified. Through this case, reasons for unsuccessful matching are explored. As shown in two unmatched subvascular trees in Figure 5, significant bending can be seen, possibly due to surgical suturing or lung collapse. Our matching algorithm, based on similarity, uses morphological dilation to overcome slight bending. However, severe bending still poses challenges in determining their correspondence. The other set of unmatched subvascular trees represents a shorter segment. The reason for not matching, similar to the discussion in Section 3.1.1. about the second phase of experiments in lung regions without lesions, is that this structure did not appear in the pre-operative vascular segmentation and thus could not be matched. Although these three sets of subvascular trees were not successfully matched, potentially affecting the image registration results, other successfully matched subvascular trees, generating at least 1000 pairs of control points evenly distributed throughout the surgical lung region and covering the areas near the surgical site, provide sufficient control points for the final image registration to still be achievable for this set of images.

### 3.2. Target Registration Errors

Target registration error is employed as an evaluation metric to compare the outcomes of completed localization, the method proposed by Stavropoulou et al. [24] (referred to hereafter as the comparative continuous method), and our proposed approach. To further compare these methods, a paired sample t-test was conducted on the latter two approaches. Additionally, 20 sets of target points were selected for analysis in both the left and right lungs, including 20 pairs of points each from lung regions without lesions and surgical lung regions.

#### 3.2.1. Experiment on Lung Regions without Lesions

Table 3 displays the experimental results for lung regions without lesions. It was found that after localization, about half of the cases achieved better alignment results ($\bar{TRE}\leq 2.50$). This suggests that the main image differences in lung regions without lesions before and after surgery may stem from variations in patient positioning and respiratory phases in the images. Therefore, preliminary localization had already achieved better image registration results for some cases.

Further analysis compared the continuous method with our method regarding their effectiveness. Except for cases 4, 6, 8, and 9, there were no significant differences in the results of the two methods (p≥0.05). Particularly, case 6 was focused on because, in this instance, the comparison method ($\bar{TRE}=10.78\pm 11.10$) showed a relatively larger difference compared to the proposed method ($\bar{TRE}=1.48\pm 0.61$). It was observed that there were significant differences in the respiratory phase of this image pair, leading to poorer registration results in the lower lung region with the comparison method. After eliminating the impact of differences in the respiratory phase, both the comparison method and the proposed method achieved ideal registration results ($\bar{TRE}\leq 1.50$) for most cases. This indicates that our proposed method also performs well in lung regions without lesions and can effectively overcome differences in respiratory phases.

#### 3.2.2. Experiment on Surgical Lung Regions

Table 4 presents the comparison results of the proposed method with the comparative continuous method in the surgical lung regions. Upon analyzing the outcomes of the two approaches, it was found that significant differences exist between them in all 12 cases (*p* < 0.05), with some cases showing highly significant differences (*p* < 0.01 or *p* < 0.001). The comparative method generally underperformed in most cases, with some results being inferior even to completed localization alone. This suggests that the method may not be suitable for post-operative lung image registration and could sometimes cause unnatural expansion in certain image regions, leading to worse registration outcomes.

Turning to the outcomes obtained with our method, the target registration error for all cases was less than 2.50 mm. Although there is a slight difference compared to the results in lung regions without lesions, this still demonstrates that our method can effectively complete image registration before and after lung resection surgery, also showing good alignment performance. However, the consequences of variations in TRE between surgical and lesion-free lung regions reveal that, while registration performance is generally consistent, complex surgical deformations cause significant variations, increasing TRE in specific regions. This indicates that surgical deformations create localized discrepancies, affecting registration precision.

### 3.3. Comparison of Image Registration Methods

A method was developed using thin-plate spline functions, allowing for continuous deformation across most image areas while enabling discontinuous deformation in specific regions, such as near surgical resection areas. The key lies in the flexible selection of control points, rather than rigidly using all control points, thus breaking the continuity assumption where necessary.

**Figure 6:**
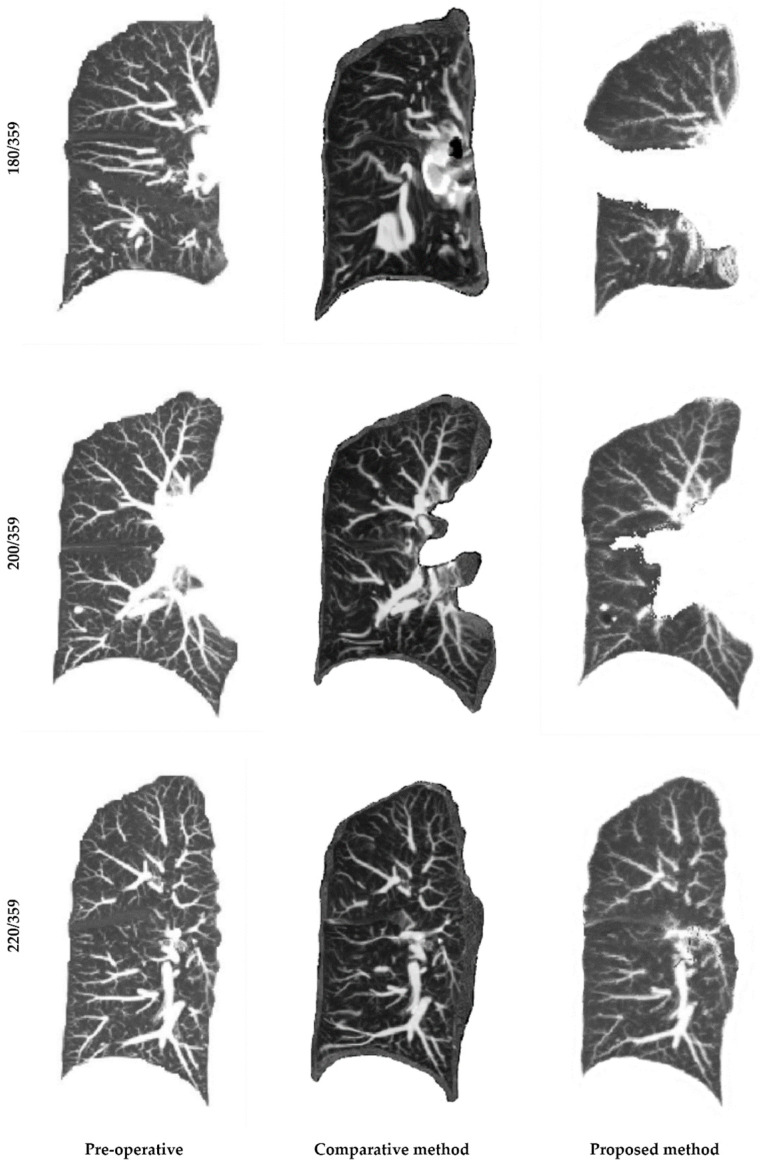
Demonstration of case 5 image registration results: The images are presented in the coronal plane, with the first column on the left displaying the pre-operative original images for reference. The second column in the middle shows the results after registration using the comparative continuous method, while the third column on the right displays the registration results of the proposed method. A total of 359 slices were included, with the top row showing slice number 180, the middle row displaying slice number 200, and the bottom row presenting slice number 220.

In the following Figure 6, pre-operative images will be shown and analyzed against both the proposed method and the approach proposed by the aforementioned researchers. For ease of observation, five image layers were superimposed and subjected to maximum intensity projection. Initially, from the results in the top first row, it is observed that the comparative method, based on the continuity assumption, failed to effectively reconstruct the surgically resected area, unnaturally enlarging surrounding areas to fill gaps. This led to image distortion and deformation, causing the vascular tree structure to not align with the pre-operative original image. In contrast, the method allowing for a degree of discontinuous deformation could more accurately reconstruct the surgically resected part, closely resembling the original image in vascular tree structure. Observing the results in the middle second row, similarities are found in the upper half of the image between the two methods, with primary differences in the lower half, especially in the lung lobe affected by surgery. Here, the comparative method showed some distortion and deformation, leading to incomplete correspondence in vascular tree structure. Lastly, in the bottom third row comparison, the overall results of the two methods do not significantly differ, but the proposed method is closer to the original image in terms of vascular tree details. However, some limitations exist, particularly in presenting lung contours, where slight discontinuities may occur. This is mainly due to the algorithm’s design allowing for the handling of discontinuous deformation, potentially leading to discontinuities at lung fissures. Nonetheless, as lung contours are not the primary observation area of the study, this issue has a relatively minor impact on the overall research.

### 3.4. Resection Margin Distance Measurement

**Figure 7:**
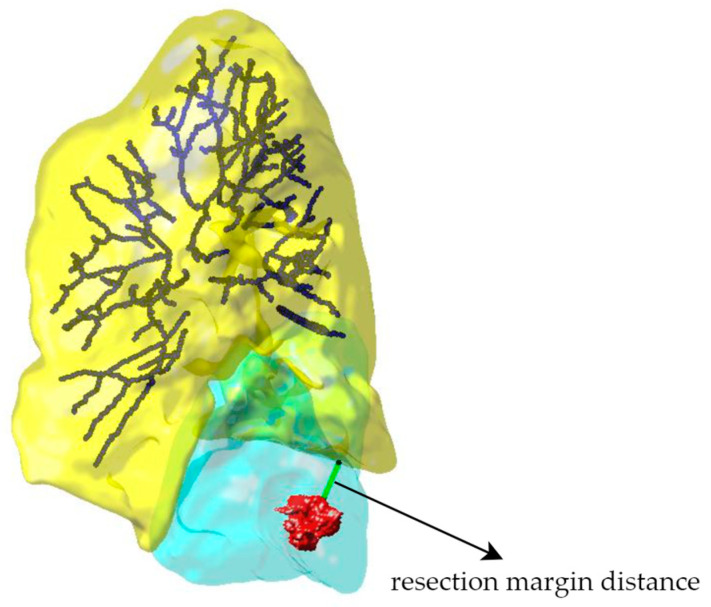
Demonstration of resection margin distance measurement: The yellow part represents the deformed post-operative lung mask, the blue part depicts the simplified presentation of the deformed post-operative vascular tree, and the red part shows the pre-operative tumor mask. The light blue indicates the reconstructed surgical resection. This case demonstrates the reconstruction result of left S9 and S10 segmentectomy. The arrow in the figure points to the resection margin distance, which is measured to be 16.09 mm.

Table 5 demonstrates the measurement results for the resection margin distance, specifically the Euclidean distance between the closest points of the resection edge and the tumor edge, as shown in Figure 7. Notably, the measured distances for cases 1 and 3 are less than the resolution of a single voxel (1 × 1 × 1 mm), hence these distances are marked as <1.00 mm.

## 4. Discussion

Low-dose CT is currently the only recommended lung cancer screening method and is commonly used for post-operative follow-up, allowing patients to have pre- and post-operative imaging for evaluating the extent of resection and reconstructing possible resection margin distances [25]. This study focuses on lung image registration, particularly considering the high deformability of lung tissue. While existing lung image registration algorithms have been applied in radiotherapy and other clinical scenarios [26,27,28,29,30,31,32], there have been no studies or applications specifically for aligning pre- and post-operative lung images. The challenge of this study lies in the various changes that may occur in the lung after surgery, such as lung collapse, local deformations caused by surgical incisions, and changes in overall lung volume. Therefore, this study will develop innovative methods to reconstruct the surgical resection area, investigate the effects of surgery on lung morphology, and use these results to accurately measure the resection margin distance. We first performed image pre-processing to standardize spatial resolution, followed by lung and tumor segmentation, addressing positional and respiratory differences between pre- and post-operative images. Our key strategy centered on segmenting lung vessels and accurately matching subvascular trees, which is essential for addressing post-operative lung deformation. We then implemented image registration using transformations based on thin-plate spline. This method is crucial for creating non-continuous deformation fields around the surgical resection area, allowing for accurate measurement of the resection margin distance and thorough assessment of the surgical range. This approach underscores the significance of defining and matching subvascular trees and utilizing TPS-based solutions to address complex deformation.

The results indicate that experiments were conducted on matching subvascular trees in lung regions without lesions and in surgical lungs. The first-stage matching experiment revealed an average completion rate of 65% for lung regions without lesions, while the rate decreased to 50% for surgical lung regions. This decline reflects the significant impact of surgical deformations on the matching results. However, the design of the two-stage subvascular tree matching algorithm still facilitates task completion. Moreover, the interpolation method based on thin-plate spline functions more accurately reconstructs the surgical resection area, bringing the vascular tree structure closer to its original state before surgery. Evaluation of the target registration error showed excellent registration results for both lesion-free and surgical lung regions, with an average registration error of less than 2.5 mm across all cases. The largest standard deviation was observed in case #7 for the surgical lung regions, at 1.65 mm, which is acceptable in our application. Particularly in the surgical lung regions, the technique demonstrated significant improvements compared to the state-of-the-art continuous method [24], which focuses on issues that are slightly similar. Compared to current continuous methods, the proposed approach addresses non-continuous deformation fields near surgical areas by generating uniformly distributed control point pairs using subvascular tree definition and matching, as well as centerline point-set registration. A subset of these pairs is strategically selected to overcome the continuity assumption in deformation fields. Ultimately, the measurement and visualization of the resection margin distance were successfully achieved.

In the application of registration technology, although our method is effective, there are some limitations. In particular, we observed possible minor discontinuities in the lung contours and fissures. For the lung contours, boundary conditions can be applied to constrain deformation, although the design of these boundary conditions still requires further discussion. Regarding the fissures, we have found that each lung lobe may have its own deformation trends and mechanical models, suggesting that the deformations between lung lobes should be considered separately in the calculations. In addition, we currently rely mainly on larger diameter lung vascular structures as references for registration. While this is effective to some extent, it overlooks finer vascular structures and image intensity information. In future work, integrating these finer vascular structures and image intensity information could be beneficial. The currently extracted subvascular tree feature points could serve as preliminary information. Designing convolutional networks to extract more matching information might help overcome the limitations of traditional algorithms.

A major and critical challenge in this research area is the absence of direct ground truth to validate surgical reconstruction outcomes and resection margin distances, relying primarily on indirect evaluation methods. Collaborating with clinical experts to directly measure resection margin distances on surgical specimens using standardized methods would provide a stronger reference base for the findings. Additionally, longitudinal studies examining the correlation between post-operative recurrence rates, survival rates, and measured resection margin distances would contribute to affirming the clinical relevance of the methodology. The proposed method only provides an anatomic evaluation by reconstructing the resected volume and cannot be used to functionally estimate the post-operative pulmonary function loss directly

## 5. Conclusions

Our method has demonstrated the capability to assess the resection margin post-sublobar resection in lung cancer patients through the comparison of 3D pre-operative and reconstructed post-operative CT images. Future research should incorporate further validation using a multicenter, large cohort and analyze its correlation with clinical outcomes.

## Figures and Tables

**Table 1:** Subvascular tree matching results in lung regions without lesions.

Case No.	Experiment Side		Total No.	Matched	Unmatched
#1	Left lung	I	25	21	4
		II	25	25	0
#2	Left lung	I	16	10	6
		II	16	16	0
#3	Left lung	I	13	10	3
		II	13	13	0
#4	Left lung	I	17	6	11
		II	17	15	2
#5	Left lung	I	21	16	5
		II	21	21	0
#6	Left lung	I	21	12	9
		II	21	19	2
#7	Left lung	I	23	17	6
		II	23	23	0
#8	Left lung	I	21	12	9
		II	21	20	1
#9	Right lung	I	27	20	7
		II	27	27	0
#10	Left lung	I	17	9	8
		II	17	16	1
#11	Right lung	I	20	17	3
		II	20	20	0
#12	Left lung	I	19	14	5
		II	19	18	1

**Table 2:** Subvascular tree matching results in surgical lung regions.

Case No.	Operative Location		Total No.	Matched	Unmatched
#1	RML ^1^	I	18	13	5
		II	18	18	0
#2	RML ^1^	I	17	12	5
		II	17	15	2
#3	RML ^1^	I	17	10	7
		II	17	16	1
#4	RML ^1^	I	15	10	5
		II	15	15	0
#5	R (S6) ^2^	I	16	7	9
		II	16	14	2
#6	R (S6) ^2^	I	19	7	12
		II	19	16	3
#7	R (S7, 8, 9, 10) ^2^	I	13	5	8
		II	13	11	2
#8	L (S6) ^2^	I	17	6	11
		II	17	15	2
#9	L (S9, 10) ^2^	I	20	8	12
		II	20	20	0
#10	R (S8) ^2^	I	15	5	10
		II	15	13	2
#11	LUL ^3^	I	26	13	13
		II	26	24	2
#12	RUL ^3^	I	24	8	16
		II	24	23	1

^1^ Cases undergoing lobectomy. ^2^ Cases undergoing segmentectomy. ^3^ Cases undergoing wedge resection.

**Table 3:** Target registration error results in lung regions without lesions.

		Target Registration Error (mm)		
Case No.	Method	M ± SD (S)	Max	Min
#1	After localization	1.54 ± 0.73	3.33	0.38
	Comparative continuous method	1.06 ± 0.50	2.02	0.13
	Proposed method	1.09 ± 0.43 (ns)	2.16	0.13
#2	After localization	2.09 ± 1.01	4.53	0.57
	Comparative continuous method	1.05 ± 0.43	2.18	0.33
	Proposed method	0.99 ± 0.46 (ns)	2.14	0.21
#3	After localization	2.05 ± 1.30	4.66	0.27
	Comparative continuous method	1.08 ± 0.51	2.44	0.25
	Proposed method	1.08 ± 0.44 (ns)	2.01	0.44
#4	After localization	1.94 ± 0.77	3.31	0.36
	Comparative continuous method	1.43 ± 0.72	3.05	0.41
	Proposed method	1.11 ± 0.61 *	2.66	0.38
#5	After localization	22.99 ± 3.17	36.96	24.53
	Comparative continuous method	1.31 ± 0.58	2.92	0.36
	Proposed Method	1.19 ± 0.42 (ns)	2.07	0.50
#6	After localization	6.62 ± 2.69	10.28	1.60
	Comparative continuous method	10.78 ± 11.10	28.42	0.67
	Proposed method	1.48 ± 0.61 ***	2.82	0.67
#7	After localization	4.95 ± 2.21	10.10	1.52
	Comparative continuous method	2.80 ± 4.60	16.66	0.41
	Proposed method	1.34 ± 0.61 (ns)	2.33	0.17
#8	After localization	6.91 ± 1.66	9.58	3.36
	Comparative continuous method	1.14 ± 0.53	2.60	0.35
	Proposed method	0.90 ± 0.47 **	2.89	0.39
#9	After localization	2.33 ± 1.67	7.84	0.55
	Comparative continuous method	1.30 ± 0.58	2.36	0.22
	Proposed method	0.96 ± 0.61 **	2.90	0.15
#10	After localization	5.76 ± 1.31	8.67	4.22
	Comparative continuous method	1.33 ± 0.47	2.05	0.30
	Proposed method	1.50 ± 0.76 (ns)	3.03	0.60
#11	After localization	17.42 ± 2.71	21.69	10.81
	Comparative continuous method	2.19 ± 1.11	5.59	0.75
	Proposed method	1.62 ± 0.82 ***	3.22	0.33
#12	After localization	4.86 ± 2.18	9.09	1.60
	Comparative continuous method	2.21 ± 0.98	5.46	0.79
	Proposed method	1.53 ± 0.56 ***	2.77	0.35

* *p* < 0.05. ** *p* < 0.01. *** *p* < 0.001. ns: not significant (*p* > 0.05).

**Table 4:** Target registration error results in surgical lung regions.

		Target Registration Error (mm)		
Case No.	Method	M ± SD (S)	Max	Min
#1	After localization	7.54 ± 6.29	28.39	0.67
	Comparative continuous method	6.11 ± 10.87	41.21	0.36
	Proposed method	1.68 ± 1.36 *	5.85	0.20
#2	After localization	8.04 ± 5.81	23.20	1.31
	Comparative continuous method	7.35 ± 13.57	52.28	0.19
	Proposed method	1.51 ± 0.83 *	3.18	0.42
#3	After localization	6.82 ± 5.39	16.38	0.78
	Comparative continuous method	5.23 ± 7.61	22.47	0.42
	Proposed method	1.11 ± 0.42 *	2.29	0.48
#4	After localization	10.20 ± 3.92	19.12	3.60
	Comparative continuous method	5.04 ± 6.72	19.54	0.43
	Proposed method	1.44 ± 1.09 **	4.40	0.29
#5	After localization	23.26 ± 8.02	36.28	7.23
	Comparative continuous method	16.25 ± 11.40	37.38	0.39
	Proposed Method	2.16 ± 1.48 ***	5.83	0.24
#6	After localization	15.80 ± 5.00	28.34	4.07
	Comparative continuous method	33.19 ± 14.09	66.69	3.56
	Proposed method	1.72 ± 1.05 ***	4.25	0.24
#7	After localization	12.73 ± 2.92	17.95	8.10
	Comparative continuous method	8.65 ± 11.47	39.42	0.44
	Proposed method	2.35 ± 1.65 *	6.20	0.22
#8	After localization	11.62 ± 03.41	17.52	5.60
	Comparative continuous method	16.72 ± 8.61	26.69	0.51
	Proposed method	1.53 ± 1.35 **	6.45	0.25
#9	After localization	11.40 ± 5.05	26.31	6.10
	Comparative continuous method	2.34 ± 3.15	11.83	0.30
	Proposed method	1.05 ± 0.90 *	3.15	0.31
#10	After localization	12.17 ± 3.68	18.80	5.89
	Comparative continuous method	4.73 ± 5.46	14.99	0.72
	Proposed method	1.40 ± 0.90 *	3.35	0.20
#11	After localization	18.18 ± 4.18	28.98	8.62
	Comparative continuous method	3.13 ± 4.67	22.80	0.75
	Proposed method	1.27 ± 0.52 ***	2.92	0.63
#12	After localization	22.94 ± 7.71	40.33	11.98
	Comparative continuous method	33.98 ± 15.22	66.25	2.15
	Proposed method	1.98 ± 1.32 ***	4.84	0.37

* *p* < 0.05. **: *p* < 0.01. *** *p* < 0.001.

**Table 5:** Resection margin distance measurement results.

Case No.	Surgical Procedure/Location	Margin Distance (mm)
#1	Lobectomy/RML	<1.00
#2	Lobectomy/RML	4.69
#3	Lobectomy/RML	<1.00
#4	Lobectomy/RML	11.87
#5	Segmentectomy/R (S6)	4.12
#6	Segmentectomy/R (S6)	6.16
#7	Segmentectomy/R (S7, 8, 9, 10)	5.39
#8	Segmentectomy/L (S6)	5.74
#9	Segmentectomy/L (S9, 10)	16.09
#10	Segmentectomy/R (S8)	1.73
#11	Wedge resection/LUL	6.16
#12	Wedge resection/RUL	13.08

## Data Availability

All data generated or analyzed during this study are included in this published article.
